# Supplementary material for: Higher rank chirality and non-Hermitian skin effect in a topolectrical circuit
Source: Nat Commun. 2023 Feb 9;14:720. doi: 10.1038/s41467-023-36130-x (PMC9911780; doi:10.1038/s41467-023-36130-x)
Supplement: Supplementary file 1 — Supplementary Information [file 41467_2023_36130_MOESM1_ESM.pdf]

Supplementary Materials: Higher rank chirality and momentum resolved non-Hermitian skin effect in a topolectrical circuit

Penghao Zhu[1], Xiao-Qi Sun[1], Taylor L. Hughes[1*], and Gaurav Bahl[2*]

[1]Department of Physics and Institute for Condensed Matter Theory
[2]Department of Mechanical Science and Engineering,, University of Illinois
Urbana-Champaign, Urbana, Illinois 61801, USA
[*]To whom correspondence should be addressed: hughest@illinois.edu, bahl@illinois.edu

December 17, 2022

S1 Dynamics of long-lived modes and the NHSE

Here we discuss the relationship between the dynamics of long-lived modes and the NHSE in 1D non-Hermitian systems. In Ref. 1, the authors proved that the point gap winding number around a reference point E_0 in the complex energy plane, $W_1(E_0)$, equals the chiral imbalance of modes with real energy $\mathrm{Re}\, E_0$, i.e.,

$$W_1(E_0) = \sum_i \mathrm{sgn}\left(\mathrm{Im}(E_i - E_0)\right) \chi_i, \tag{S1}$$

where χ_i and E_i are the chirality and complex energy of the i-th mode with real energy $\mathrm{Re}\, E_0$, and the sum is over all modes with real energy $\mathrm{Re}\, E_0$. This equation suggests that the net chirality of long-lived modes (i.e., those with $\mathrm{Im}(E_i - E_0) > 0$) determines the sign of the point-gap winding number. The chirality of a 1D mode is just the sign of its group velocity, which captures the dynamics of this mode. Meanwhile, Ref. 2 shows that the sign of the point-gap winding number determines on which boundary the skin modes localize. Thus, we can draw a conclusion that the net chirality of long-lived modes tells us where the skin modes localize. Specifically, for a reference energy E_0 such that $\mathrm{Im}(E_i - E_0) > 0$ only for the slowest decay mode, the sign of the point-gap

winding number is determined by the chirality of the slowest decay mode equals, and thus the dynamics of the slowest decay mode determines the localization of the skin modes.

S2 Topology related to the rank-2 NHSE

In order to understand the topology related to the rank-2 NHSE, let us review NH point-gap topology and the NHSE in 1D. Given a translation invariant NH Hamiltonian we can Fourier transform to arrive at a NH Bloch Hamiltonian $H(\mathbf{k})$. One can define a point gap of $H(\mathbf{k})$ at a complex energy E if its spectrum does not cross E, i.e., if $\det(H(\mathbf{k})-E) \neq 0$. This means that $\det(H(\mathbf{k})-E)$ is a non-zero complex function of $\mathbf{k}$, and has a well-defined winding number $W_1(E)$ in the 1D Brillouin zone (BZ) protected by the point gap at E:

$$W_1(E) = -\int_0^{2\pi} \frac{dk}{2\pi} \frac{\partial}{\partial k} \arg\left[\det\left(H(k) - E\mathbb{1}\right)\right]. \tag{S2}$$

Furthermore, it has been shown that $W_1(E) \in \mathbb{Z}$ counts the number of eigenstates at energy E that are localized at a boundary of a semi-infinite chain [2]. Since $W_1(E)$ can be nonzero for a continuous region of the complex energy plane (bounded by point-gap closing points), there can be an extensive number of eigenstates localized on the boundary, i.e., there is a NHSE. One simple example is the Hatano-Nelson chain with Bloch Hamiltonian

$$H_{\mathrm{HN}} = t\sin k + i(g\cos k + E_0), \tag{S3}$$

of which the spectrum forms a loop in the complex energy plane as shown in Fig. S1. When k goes from 0 to 2π, the spectrum winds any point inside the loop once, and thus has the winding number $W_1 = 1$ and NH skin modes localized on the right boundary.

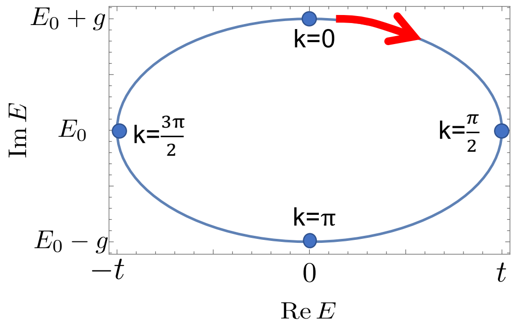

Figure S1: Spectrum of the Hatano-Nelson chain. The red arrows indicate the nontrivial winding of E when we change k.

For our model with Bloch Hamiltonian $H(\mathbf{k}) = \sin k_x \sin k_y + i(\cos k_x + \cos k_y - m)$ [c.f. Eq. (5) of the main text], at each given k_x (k_y), it is actually a Hatano-Nelson chain along $y-$ ($x-$) direction. Thus, we can define a

1D point-gap winding number at each k_x (k_y) following Eq.(S2), and derive the momentum-resolved 1D point gap winding number, $W_1(E, k_x)$ ($W_1(E, k_y)$). The the sign of $W_1(E, k_x)$ ($W_1(E, k_y)$) is determined by the sign of $\sin k_x$ ($\sin k_y$), and therefore corresponds to the momentum-resolved skin effect observed under a cylinder geometry where y- (x-) direction is open but x- (y-) direction is periodic. However, for the NHSE under square geometry, we can only say that the edge- and corner- localized skin modes are closely related to $W_1(E, k_x)$ and $W_1(E, k_y)$ because intuitively $(W_1(E, k_y), W_1(E, k_x)) = (\text{sgn}(k_y), \text{sgn}(k_x)) = (\text{sgn}(v_x), \text{sgn}(v_y))$ where (v_x, v_y) is group velocity of the long-lived rank-2 modes (we choose k_x and k_y in the range of $(-\pi, \pi]$). However, we have not rigorously proved the correspondence, and leave the discussion about the possible correspondence between rank-2 NHSE and point-gap topology into future research. Actually, the origin of 2D NHSE effects is still an open question, and our model can serve as an interesting case for future study.

The above discussion can be supported by spectrum of Eq. (5) under a torus geometry as shown in Fig. S2 (a): We indeed see lots of loops that corresponds to nonzero $W_1(E, k_x)$ and $W_1(E, k_y)$. One example loop is emphasized by a blue oval in Fig. S2 (a), which is two-fold degenerate because $k_x = \pm k_{x0}$ corresponds to the same loop.

Though momentum-resolved 1D point-gap winding in the spectrum under a torus geometry is well understood, the spectrum under a cylinder and a square geometry show some mysterious behaviors. Under cylinder geometry where one direction is periodic and another direciton is open, most eigenstates have their energy on the imaginary axis as predicted by the generalized Brillouin zone theory [Fig. S2 (b)] However, there are some other states that have complex energy forming loops in the complex energy plane which cannot be understood from the canonical generalized Brillouin zone theory, which bears further investigations. Surprisingly, under the square geometry where both directions are open, the spectrum suddenly change from a line on the imaginary axis to a region in the complex energy plane [Fig. S2 (c)]. This phenomenon has not been observed in any other previously known systems (including the system in Ref. 18), and needs better understandings in future research.

S3 Review of topolectric circuit theory

We present here a brief review of the theoretical foundation for topolectric circuits, based on the extensive discussion that can be found in Ref. 3. The behavior of any passive circuit with only resistors, capacitors, and inductors is governed completely by Kirchhoff's and Ohm's laws. The total input current into a node equals the total outgoing current from that node:

$$I_a = \sum_j I_{aj} + I_{aG}, \tag{S4}$$

where I_a is the total input current into node a, and I_{aj} is the current flowing from node a to an adjacent (directly connected) node j, and I_{aG} is the current

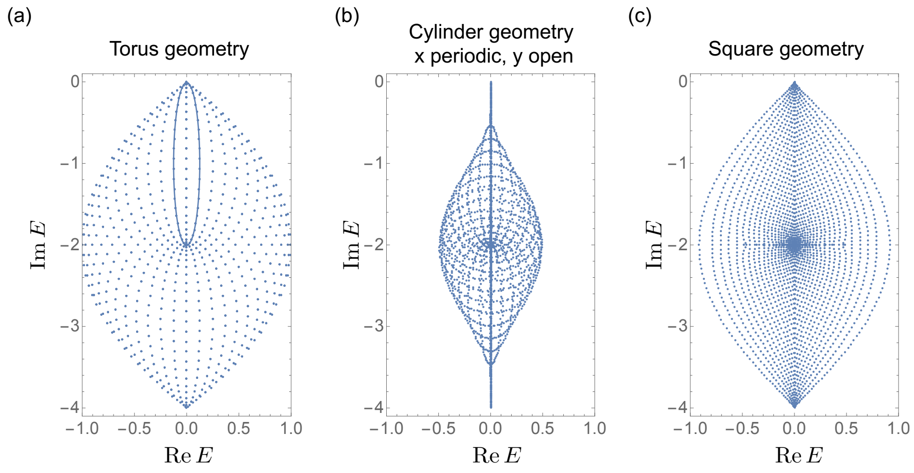

Figure S2: The spectra of our model with $m = 2$ under torus, cylinder, and square geometry.

flowing from node a to the ground. The relation between the currents and voltages in the network is:

$$\dot{I}_{aj} = C_{aj}\left(\ddot{V}_a - \ddot{V}_j\right) + \frac{1}{R_{aj}}\left(\dot{V}_a - \dot{V}_j\right) + \frac{1}{L_{aj}}\left(V_a - V_j\right), \tag{S5}$$

where the dot indicates a time derivative; V_i is the voltage of node i; C_{aj}, R_{aj}, and L_{aj} are capacitors, resistors, and inductors between node a and node j. If we Fourier transform Eq. S4 and Eq. S5, we obtain two frequency-dependent equations:

$$\begin{aligned} I_a(\omega) &= \textstyle\sum_j I_{aj}(\omega) + I_{aG}(\omega), \\ I_{aj}(\omega) &= \left(i\omega C_{aj} + \tfrac{1}{R_{aj}} + \tfrac{1}{i\omega L_{aj}}\right)\left(V_a(\omega) - V_j(\omega)\right), \end{aligned} \tag{S6}$$

where ω is the angular frequency. From Eq. S6, we can write the relation between the input currents and output voltages as

$$I_a(\omega) = J_{ab} V_b(\omega), \tag{S7}$$

where $I = (I_1\ I_2\ \ldots)^T$, $V = (V_1\ V_2\ \ldots)^T$, and J is the circuit Laplacian matrix with entries

$$\begin{aligned} J_{aj} &= -\left(i\omega C_{aj} + \tfrac{1}{R_{aj}} + \tfrac{1}{i\omega L_{aj}}\right) \\ J_{aa} &= \left(i\omega C_{aG} + \tfrac{1}{R_{aG}} + \tfrac{1}{i\omega L_{aG}}\right) + \textstyle\sum_{j\neq a}\left(i\omega C_{aj} + \tfrac{1}{R_{aj}} + \tfrac{1}{i\omega L_{aj}}\right), \end{aligned} \tag{S8}$$

where C_{aG}, R_{aG}, and L_{aG} are capacitors, resistors, and inductors between node a and the ground. By construction, J is a symmetric matrix.

The core idea behind topoletric circuits is to realize a tight-binding Hamiltonian using the circuit Laplacian. In other words, given a symmetric tight-binding Hamiltonian H in real space (or equivalently a reciprocal Hamiltonian

in momentum space, i.e, $H(\mathbf{k}) = H^T(-\mathbf{k})$), we can find a circuit Laplacian J such that $H = iJ$, and J_{aj} can be identified as the real space hoppings between sites a and sites j in the Hamiltonian H. If we want to find a topoletric circuit realization of a non-symmetric H, then we need to include more components besides resistors, capacitors, and inductors.

Using the general theory reviewed above, we can identify the corresponding circuit Laplacian for Hamiltonian $H(\mathbf{k}) = \sin k_x \sin k_y + i(\cos k_x + \cos k_y - m)$. We first Fourier transform the Bloch Hamiltonian into real space to get the real space hoppings plotted in Fig. 2a of the main text:

$$\begin{aligned} H = &\sum_{x,y} -\frac{1}{4} c^\dagger_{x,y} c_{x+1,y+1} + \frac{1}{4} c^\dagger_{x,y+1} c_{x+1,y} + h.c. \\ &+ i \sum_{x,y} \frac{1}{2} c^\dagger_{x+1,y} c_{x,y} + \frac{1}{2} c^\dagger_{x,y+1} c_{x,y} + h.c., \end{aligned} \tag{S9}$$

We want to design a circuit such that its Laplacian satisfies $J = -isH$, where s is a scale factor. As shown in Fig. 2a of the main text, we use capacitors C and inductors L to realize the two digonal hoppings with opposite sign, and use resistors R to realize hoppings along x and y directions, and the R, L, and C are selected to satisfy $\omega C : \frac{1}{\omega L} : \frac{1}{R} = 1 : 1 : 2$ for a selected frequency (4.95 kHz).

S4 Momentum information encoded in the edge-localized modes

In Fig. S3 we take a closer look at the phase information associated with the two eigenstates localized on oppposite boundaries (labeled with the green disk and square in Fig. 3a of the main text). We observe that the adjacent nodes on the top (or bottom) edge have a very clear and consistent relative phase difference of $\delta\phi = \pi/2$ (or $\delta\phi = -\pi/2$). This can be interpreted as momentum $k_x = \pi/2$ (or $k_x = -\pi/2$). A similar analysis can be readily performed on the other eigenmodes (not presented for brevity) to confirm that modes with opposite momentum localize on opposite edges, which is the expected momentum resolved NHSE.

S5 Effective local impedance of the circuit with respect to excitations with different momenta

Let us consider a cylindrical geometry where the x-direction is periodic and y-direction is open. We can perform an explicit circuit analysis to show that

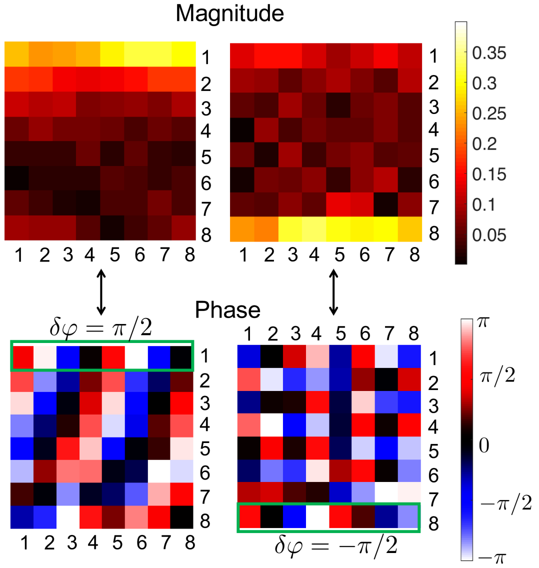

Figure S3: Magnitude and phase of edge-localized eigenstates. The top and bottom rows show the magnitude and phase for the two representative edge-localized eigenstates identified in Fig. **??**a. The value of $\delta\phi$ for the rows highlighted in green indicates the relative phase difference between the $n+1$-th and n-th nodes on the boundary.

the effective local impedances experienced by exitations on the open edge row are identical with respect to $\pm k_x$ excitations (assuming a semi-infinite circuit). To do this, we solve for the current response to an applied voltage input with frequency ω with momentum k_x on the open edge row. Because of translation symmetry along x-direction, the effective local impedance of nodes within any row will be identical, and so we only need to focus on one column and its neighbors (see Fig. S4a).

From Kirchhoff's and Ohm's laws we have the relation:

$$\begin{aligned} I_2 =& \frac{V_2 - V_1}{R} + \frac{V_2 - V_3}{R} + \frac{V_2 - V_5}{R} + \frac{V_2}{R} + V_2(i\omega C + \frac{1}{i\omega L}) \\ &+ i\omega C(V_2 - V_4) + \frac{V_2 - V_6}{i\omega L}, \end{aligned} \tag{S10}$$

where V_j is the voltage of node j, and I_j is the net current flow into node j, for excitation at frequency ω. Note that in our system $i\omega C + 1/(i\omega L) = 0$ and $2\omega C = 2/(\omega L) = 1/R$ at the frequency of interest. If we have a plane wave voltage (i.e., $V_{1n} = \exp(ik_x n)$) input on the first row, given the translation symmetry along x direction, the voltage on the j-th row should also take the

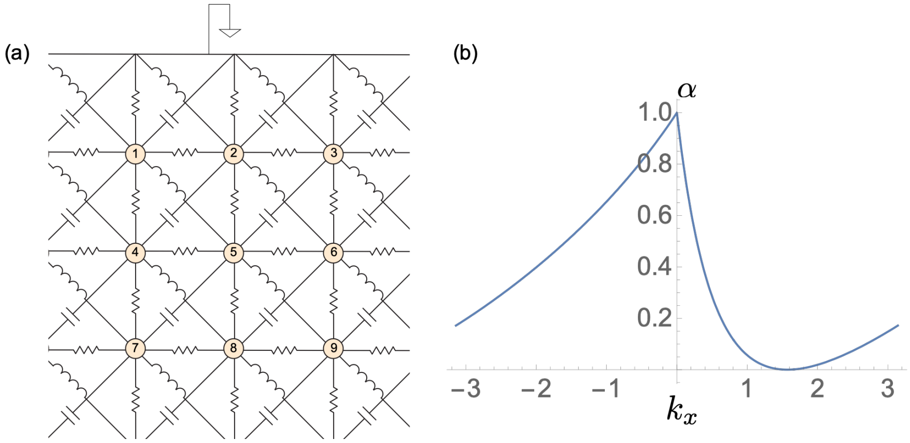

Figure S4: Circuit analysis of response to a boundary voltage input. **(a)** A part of our circuit used in the circuit analysis. **(b)** The ratio between voltage magnitude between the j-th row and (j+1)-th row.

140 form $V_{jn} = A_j \exp(ik_x n)$. Then, the above equation becomes

$$\begin{aligned} I_2 &= V_2 \left(\frac{4}{R} - \frac{1}{R} \exp(-ik_x) - \frac{1}{R} \exp(ik_x) - \frac{1}{R} \frac{V_5}{V_2} - i\omega C \frac{V_4}{V_2} - \frac{1}{i\omega L} \frac{V_6}{V_2} \right) \\ &= V_2 \left(\frac{4}{R} - \frac{2}{R} \cos k_x - \frac{\alpha_1}{R} - i\omega C \alpha_1 e^{-ik_x} - \frac{\alpha_1 e^{ik_x}}{i\omega L} \right) \\ &= V_2 \left(\frac{4}{R} - \frac{2}{R} \cos k_x - \frac{\alpha_1}{R} - \alpha_1 (\omega C + \frac{1}{\omega L}) \sin k_x \right) \\ &= V_2 \left(\frac{4}{R} - \frac{2}{R} \cos k_x - \frac{\alpha_1}{R} (1 + \sin k_x) \right), \end{aligned} \tag{S11}$$

141 where $\alpha_1 = A_2/A_1$.Thus, the effective local impedance is

$$Z = \frac{V_2}{I_2} = \frac{R}{4 - 2\cos k_x - \alpha_1 (1 + \sin k_x)}, \tag{S12}$$

142 where $\alpha_1 \equiv \alpha_1(k_x)$. More generally, we define $\alpha_n = A_{n+1}/A_n$. Let us now

calculate α_n.

$$
\begin{aligned}
&I_5 = 0 \\
&= \frac{V_5 - V_2}{R} + \frac{V_5 - V_4}{R} + \frac{V_5 - V_6}{R} + \frac{V_5 - V_8}{R} + \frac{V_5 - V_1 + V_5 - V_9}{i\omega L} \\
&+ i\omega C(V_5 - V_3 + V_5 - V_7) \\
&= V_5 \bigg(\frac{4}{R} - \frac{1}{\alpha_1 R} - \frac{2}{R}\cos k_x - \frac{\alpha_2}{R} - \frac{\frac{1}{\alpha_1}e^{-ik_x} + \alpha_2 e^{ik_x}}{i\omega L} \\
&- i\omega C(\frac{1}{\alpha_1}e^{ik_x} + \alpha_2 e^{-ik_x}) \bigg) \\
&= V_5 \bigg(\frac{4}{R} - \frac{1}{\alpha_1 R} - \frac{2}{R}\cos k_x - \frac{\alpha_2}{R} - \frac{-i\frac{1}{\alpha_1}\sin k_x + i\alpha_2 \sin k_x}{i\omega L} \\
&- i\omega C(i\frac{1}{\alpha_1}\sin k_x - i\alpha_2 \sin k_x) \bigg) \\
&= V_5 \bigg(\frac{4}{R} - \frac{1}{\alpha_1 R} - \frac{2}{R}\cos k_x - \frac{\alpha_2}{R} + \frac{1}{\alpha_1}(\frac{1}{\omega L} + \omega C)\sin k_x \\
&- \alpha_2(\frac{1}{\omega L} + \omega C)\sin k_x \bigg),
\end{aligned} \tag{S13}
$$

which indicates that

$$4 - 2\cos k_x - \frac{1}{\alpha_1}(1 - \sin k_x) - \alpha_2(1 + \sin k_x) = 0. \tag{S14}$$

Since our circuit also has a translation symmetry along y-direction, the above equation is true for any α_n and α_{n+1}:

$$4 - 2\cos k_x - \frac{1}{\alpha_n}(1 - \sin k_x) - \alpha_{n+1}(1 + \sin k_x) = 0. \tag{S15}$$

In a semi-infinite geometry, we have all α_n equal to α, because of the translation symmetry along the y-direction. Thus the above equation becomes

$$\alpha^2(1 + \sin k_x) - \alpha(4 - 2\cos k_x) + (1 - \sin k_x) = 0, \tag{S16}$$

from which we can solve

$$\alpha_\pm = \frac{2 - \cos k_x \pm 2\sqrt{1 - \cos k_x}}{(1 + \sin k_x)}. \tag{S17}$$

We note that we should choose α_- because when $k_x = \pi/2$, we expect α to be less than one, thus the α_- solution physically makes sense. Finally we get

$$\alpha(k_x) = \frac{2 - \cos k_x - 2\sqrt{1 - \cos k_x}}{(1 + \sin k_x)}, \tag{S18}$$

which is plotted in Fig. S4b. Note that the singular point at $k_x = -\pi/2$ is removable in the sense that $\lim_{k_x \to -\pi/2} \alpha(k_x) = 1/2$.

From Fig. S4b, we clearly see α is asymmetric for $\pm k_x$, which manifests the momentum resolved skin effect. However, if we substitute $\alpha_1 = \alpha$ into Eq. S12, we get

$$Z = \frac{R}{2 - \cos k_x + 2\sqrt{1 - \cos k_x}}, \tag{S19}$$

which is symmetric with k_x. In other words, even though the excitation patterns are clearly unequal, the effective impedances are identical irrespective of the directionality of momentum $\pm k_x$.

A similar proof can also be applied to evaluate the effective local impedance where there are excitations on a boundary column.

S6 Complete response measurements under a square geometry

In the main text Fig. 5a-d, we only present the magnitude response to excitations with different k_x or k_y. In the figures below we present the complete response measurements including the relative phase information between all the nodes of the circuits.

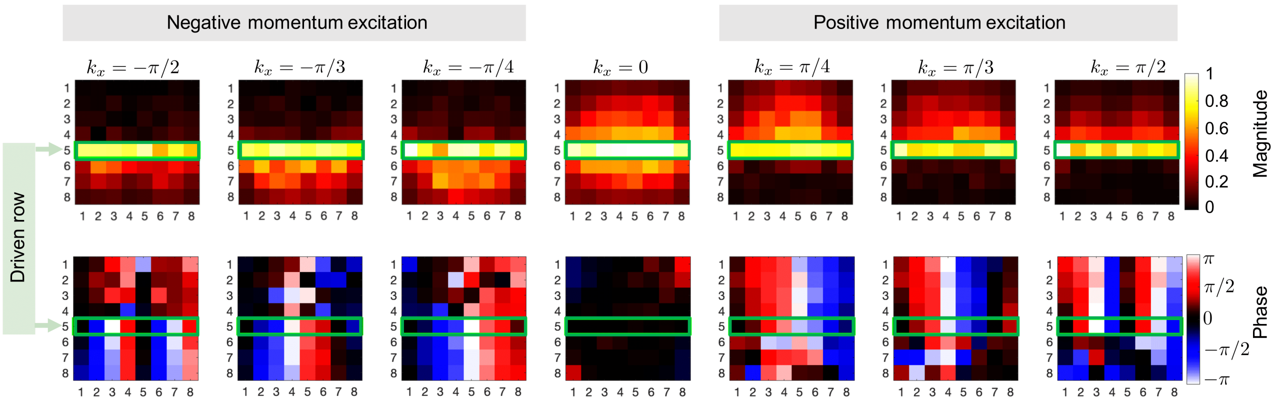

Figure S5: Responses to input excitations for the square geometry. Complete measurements of responses including both magnitude and phase information corresponding to Fig. 5a

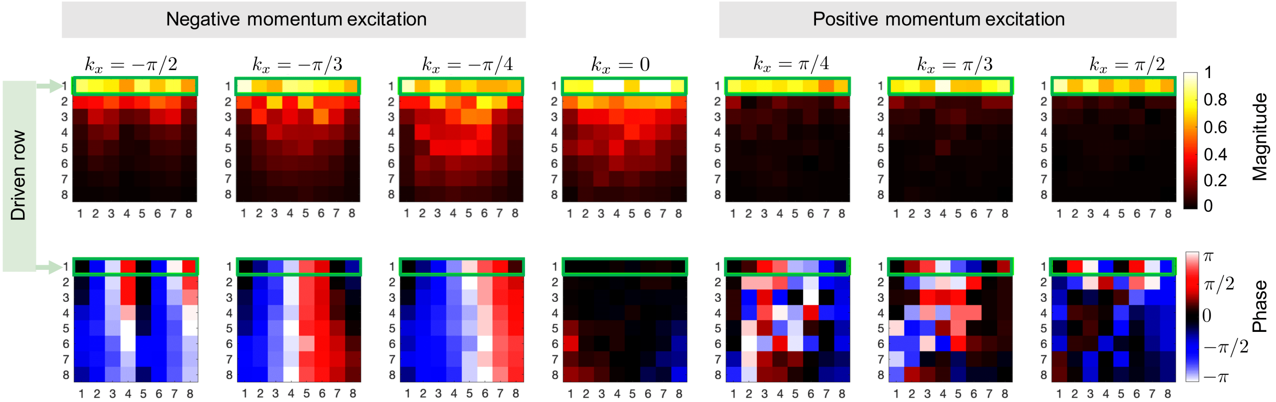

Figure S6: Responses to input excitations for the square geometry. Complete measurements of responses including both magnitude and phase information corresponding to Fig. 5b

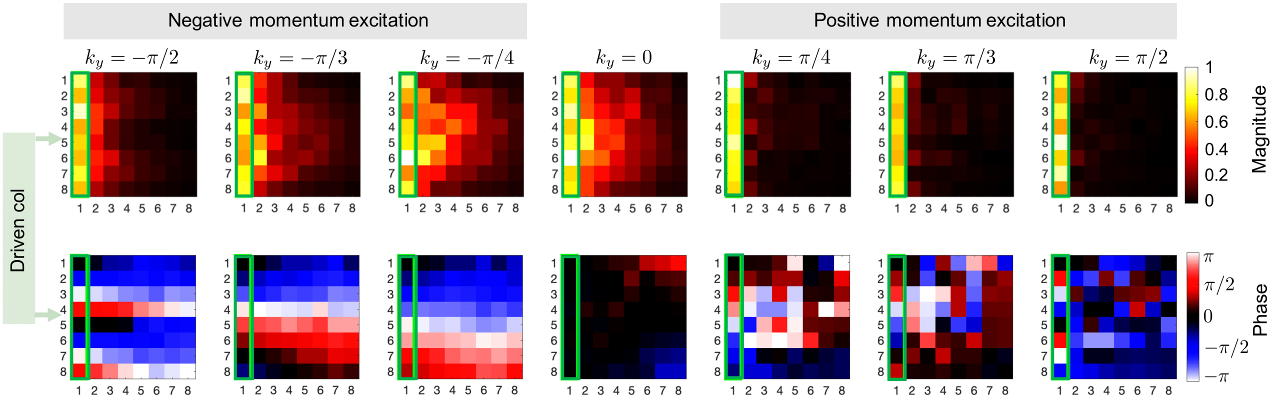

Figure S7: Responses to input excitations for the square geometry. Complete measurements of responses including both magnitude and phase information corresponding to Fig. 5c

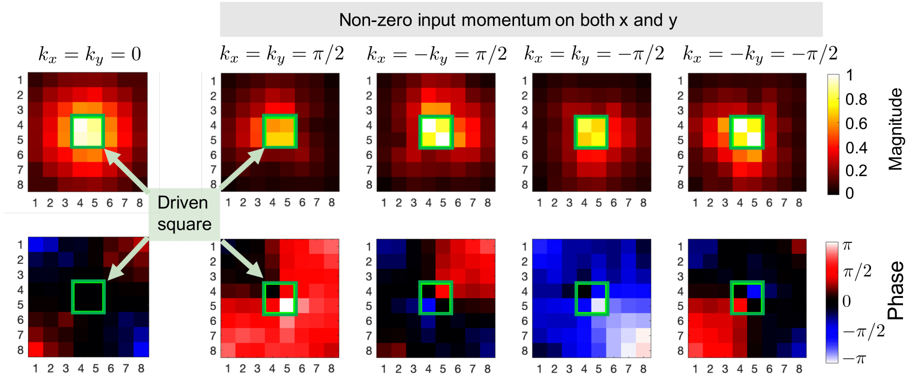

Figure S8: Responses to input excitations for the square geometry. Complete measurements of responses including both magnitude and phase information corresponding to Fig. 5d

References

[1] Bessho, T. & Sato, M. Nielsen-ninomiya theorem with bulk topology: Duality in floquet and non-hermitian systems. *Phys. Rev. Lett.* **127**, 196404 (2021).

[2] Okuma, N., Kawabata, K., Shiozaki, K. & Sato, M. Topological origin of non-hermitian skin effects. *Phys. Rev. Lett.* **124**, 086801 (2020).

[3] Lee, C. H. *et al.* Topolectrical circuits. *Communications Physics* **1**, 1–9 (2018).
